# Food Web Designer: a flexible tool to visualize interaction networks

**DOI:** 10.1007/s10340-015-0686-7

**Published:** 2015-08-09

**Authors:** Daniela Sint, Michael Traugott

**Affiliations:** Mountain Agriculture Research Unit, Institute of Ecology, University of Innsbruck, Technikerstraße 25, 6020 Innsbruck, Austria

**Keywords:** Network illustration, Ecological network, Quantitative food web, Trophic chain, Pollination network, Graphical software, Connectedness web

## Abstract

Species are embedded in complex networks of ecological interactions and assessing these networks provides a powerful approach to understand what the consequences of these interactions are for ecosystem functioning and services. This is mandatory to develop and evaluate strategies for the management and control of pests. Graphical representations of networks can help recognize patterns that might be overlooked otherwise. However, there is a lack of software which allows visualizing these complex interaction networks. Food Web Designer is a stand-alone, highly flexible and user friendly software tool to quantitatively visualize trophic and other types of bipartite and tripartite interaction networks. It is offered free of charge for use on Microsoft Windows platforms. Food Web Designer is easy to use without the need to learn a specific syntax due to its graphical user interface. Up to three (trophic) levels can be connected using links cascading from or pointing towards the taxa within each level to illustrate top-down and bottom-up connections. Link width/strength and abundance of taxa can be quantified, allowing generating fully quantitative networks. Network datasets can be imported, saved for later adjustment and the interaction webs can be exported as pictures for graphical display in different file formats. We show how Food Web Designer can be used to draw predator–prey and host-parasitoid food webs, demonstrating that this software is a simple and straightforward tool to graphically display interaction networks for assessing pest control or any other type of interaction in both managed and natural ecosystems from an ecological network perspective.

**Electronic supplementary material:**

The online version of this article (doi:10.1007/s10340-015-0686-7) contains supplementary material, which is available to authorized users.

## Key message

- Ecological network approaches provide a functional understanding of pest control but it is difficult to graphically display such complex interaction networks.
- Food Web Designer is a new software which allows to draw quantitative bi- and tripartite networks and it operates with Microsoft^®^ Windows XP, Windows 7 and Windows 8.
- Food Web Designer is available free of charge and provides a straightforward tool to graphically display food webs and other types of interaction networks.

## Introduction

Over the past two decades, ecological networks, including trophic and non-trophic interactions between species, have been extensively used to understand which mechanisms drive complex communities and how this affects the functioning of ecosystems (Ings et al. 2009). This approach has also been increasingly used to better understand how pests can be controlled in their environments (Derocles et al. 2014; Tylianakis and Binzer 2014; Welch and Harwood 2014). Pests are interacting with species within the same and other trophic levels such as natural enemies (Lundgren and Fergen 2014) and their antagonists (Gómez-Marco et al. 2015) or alternative prey (Kuusk and Ekbom 2010). The control of pests, either by biological or other means, happens within this interaction networks and it is the species’ interactions which govern, directly or indirectly, how efficient the pests’ population size and behaviour can be managed (Staudacher et al. 2013). Within ecological interaction networks especially food webs proved to be useful for assessing the efficacy of pest control with regard to factors such as crop and non-crop habitat connectivity (Derocles et al. 2014), landscape structure (Macfadyen et al. 2011), or environmental change (Tylianakis and Binzer 2014). A quantitative assessment of trophic and other ecological interactions thus provides an important functional approach for developing, modelling, and evaluating pest management measures (Tixier et al. 2013). However, due to the usually large number of interactions in communities which include pestiferous species, a merely abstract description and analysis of networks can be hard to comprehend (Bohan et al. 2013). According to the principle ‘*a picture is worth a thousand words’*, graphical representation of network data is therefore desired. Depending on the nature of the data to be depicted, several stand-alone tools and packages for R (R Development Core Team 2013) are available. For example, the software ‘Pajek’ (Mrvar and Batagelj 2015) is well suited to display and analyse large networks where many nodes are connected by binary link data. However, it is limited when the strength of interactions shall be taken into account as well. The R package ‘cheddar’ (Hudson et al. 2013) allows overcoming this limitation and can also be used to analyse network data, but it is not possible to display a network taking different (trophic) levels, taxon abundances and (trophic) link strength into account. In such cases the R package ‘bipartite’ (Dormann et al. 2008) is commonly used but it can handle only two trophic levels. Furthermore, all of these tools require command line input, which makes it harder to start using them. Driven by the need to illustrate feeding interaction data within the context of pest control (and beyond), we developed this stand-alone and easy to use program that allows generating custom-made bi- or tripartite interaction graphics with a few clicks. This program aims to fill the gap that currently exists for visualizing small- to medium-sized interaction networks in an easy and quick way: it allows displaying species abundances and species’ interactions between up to three (trophic) levels considering also the strength of these interactions.

## Program features

Food web designer has an intuitive graphical interface which allows for easy inclusion of interaction network data without the need to learn a specific syntax or memorize input commands. All data can be entered directly in the corresponding windows for species and interactions data, respectively, and interaction networks can be created within a few minutes. All data and settings can be saved and re-opened as a food web project. Due to the ability to fully change all settings of an existing food web project, the graphical display of the network can be optimized easily.

Import functions for species and interactions data are implemented and allow creating also complex networks within a short period of time. Data for import are provided in semicolon delimited text files (.csv), a file format which can be easily generated and edited. All imported data are directly accessible in the program and adaptations or corrections of single values are possible at any time without the need to re-import the full dataset. The import function also allows quickly creating several interaction networks of the same layout by editing the data of abundances and interaction strength. Up to three trophic levels can be included in the network and the links connecting the taxa between the different trophic levels can be displayed as bars or triangles. The direction of the links (top-down or bottom-up) can be defined separately for each level. Colours for taxa and interaction links can be automatically assigned or selected from a standard windows dialogue providing 16 million colours. The optional scaling bars, representing the abundance of the taxa within each (trophic) level, can be adjusted to allow for a better comparison of taxa abundances between levels. For levels serving as food resources only, the standard bar view can be switched to a circle view if no abundances/quantitative measures need to be provided for each taxon. The networks can be exported as pictures in Windows Bitmap format (.bmp) or as Portable Network Graphics (.png) for subsequent use in presentations and publications. No analysis functions are implemented in Food Web Designer itself as it is developed as a visualisation tool. However, an export function provides all species and interaction data (including the assigned colours as RGB values) in a format that can be directly loaded into the R package ‘cheddar’.

A more detailed description of the functions and use of Food Web Designer 3.0 can be found in the program handbook (Suppl. 1).

Food Web Designer can be used freely with Microsoft^®^ operating systems and is available for download at no charge from http://www.uibk.ac.at/ecology/forschung/biodiversitaet.html.en. Redistribution of Food Web Designer 3.0 is granted as long as the original files are distributed unchanged and at no charge. The use of Food Web Designer has to be cited with reference to this article.

## Example datasets

### Dataset 1: pest-alternative prey-carabid trophic interaction network

**Fig. 1:**
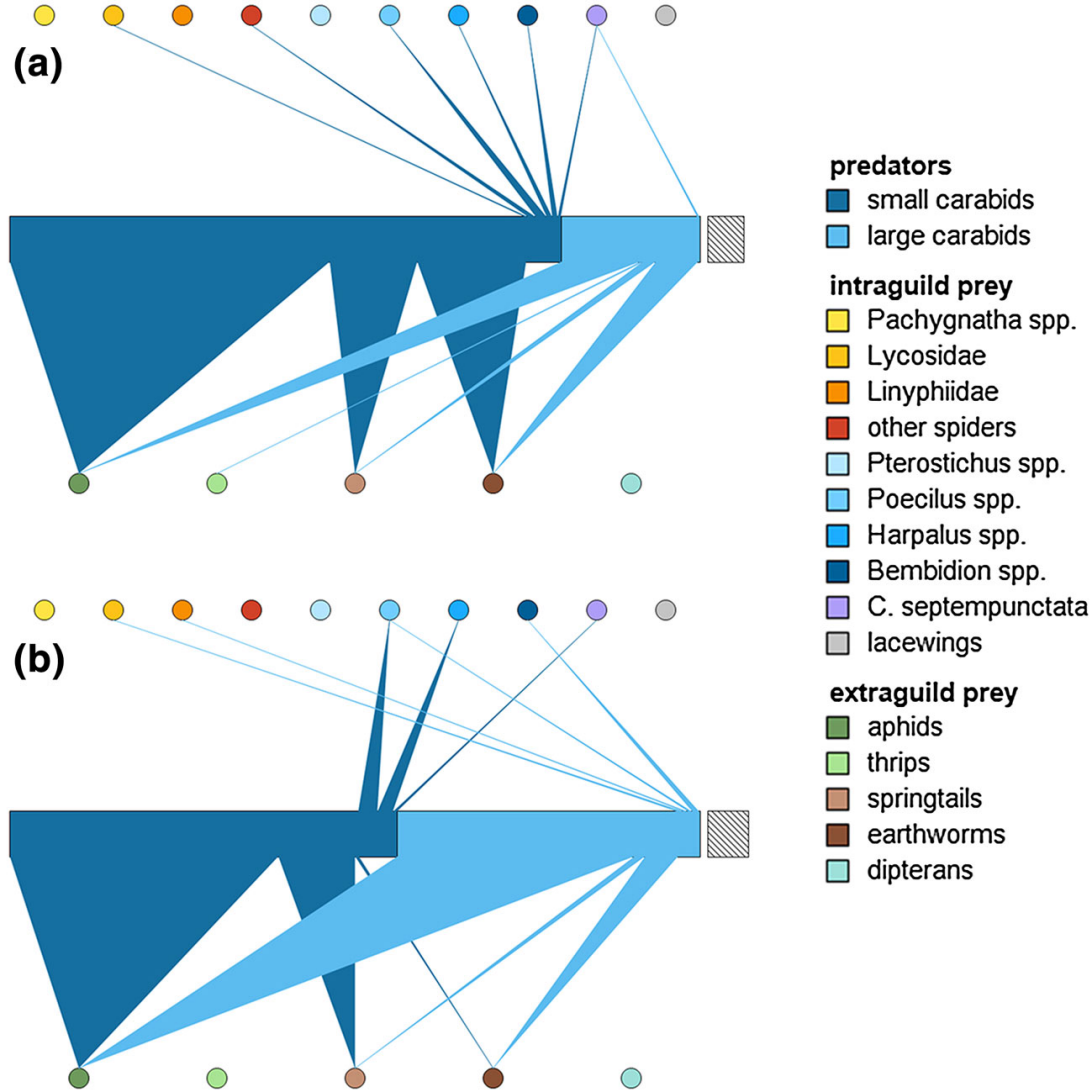
Pest-alternative prey-carabid trophic interaction network generated from data presented in Staudacher et al. (2015), illustrating the relative diet composition of small and large carabid beetles (*middle bars*) during aphid invasion (**a**) and establishment (**b**) in two barely fields. Trophic links from carabids to extraguild (*lower panel*) and intraguild (*upper panel*) prey are represented as triangles; triangle base represents the proportion of carabids testing positive for specific prey taxa. The *offset hatched bar *represents 20 (**a**) and 10 (**b**) carabid beetles, respectively

This example illustrates a trophic interaction network in aphid-infested barley fields and it is based on data presented in Staudacher et al. (2015). Carabid beetles were collected at two time points during aphid invasion and population establishment in barley fields in southern Sweden and classified as either small (<10 mm) or large (>10 mm). The carabids were then screened for the presence of prey DNA using diagnostic multiplex PCR targeting pest species (aphids, thrips), alternative extraguild (collembolans, earthworms, dipterans), and intraguild (*Pachygnatha* spp., Lycosidae, Linyphiidae, other spiders, *Pterostichus* spp., *Poecilus* spp., *Harpalus* spp., *Bembidion* spp., *Coccinellaseptempunctata*, lacewings) prey. The relative diet composition (i.e. the percentage of carabids testing positive for specific prey types) of the two carabid size classes was used to create a food web for each time point (Fig. 1). The width of the horizontal bars in the centre of the food web depicts the number of small and large carabid beetles that were molecularly tested. To separate extraguild and intraguild prey groups, the taxa classified as extraguild prey (pests and alternative prey) were positioned at the bottom of the web, while the intraguild predatory taxa were placed above the carabid consumers. Consequently, the direction of the feeding links was selected to be bottom-up in the top panel and top-down in the bottom panel. As diagnostic PCR does not allow determining the number of prey individuals consumed but provides a prey detection frequency, triangles were selected as link type. The width of the triangle base represents the proportion to which the respective prey type contributed to the relative diet composition of the consumer. As no information on the abundances of the different prey types was available, the option to display the prey taxa as circles instead of bars was selected (Fig. 1).

### Dataset 2: quantitative cereal aphid–primary–hyperparasitoid food webs

**Fig. 2:**
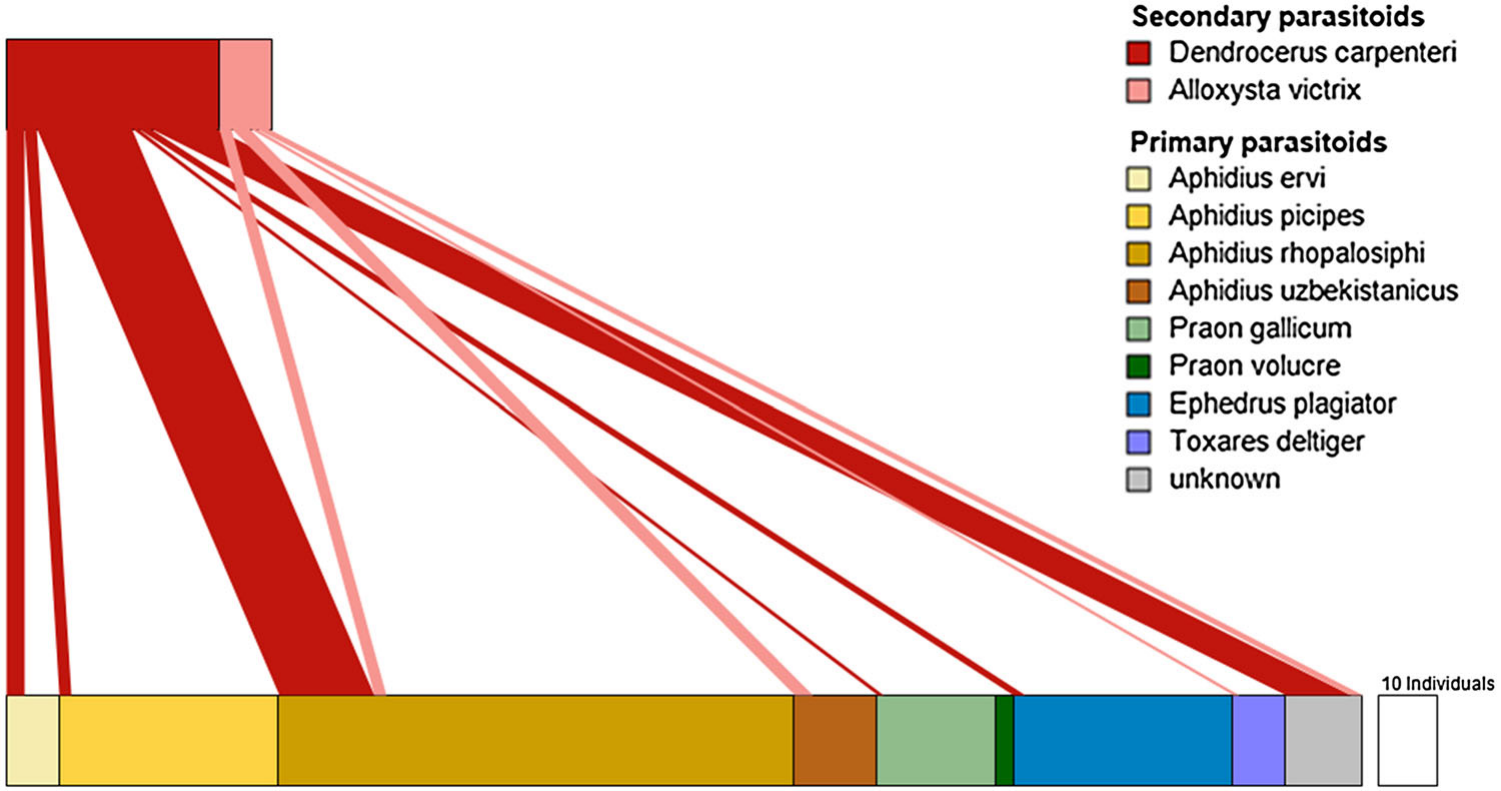
Quantitative primary parasitoid–hyperparasitoid food web generated from data presented in Traugott et al. (2008), illustrating the parasitisation of primary parasitoids of *Sitobion avenae* by two hyperparasitoid species. The *offset* *white bar* represents 10 primary and secondary parasitoid individuals, respectively

This example illustrates data presented in Traugott et al. (2008), where a hymenopteran endoparasitoid community attacking *Sitobion avenae* in a field of winter wheat was identified molecularly. The traditional approach of parasitoid rearing does not allow determining the links between primary and secondary parasitoids, as in hyperparasitized aphids only the secondary parasitoid emerges. By screening aphids and aphid mummies for the DNA of both primary and secondary parasitoids, it is possible to determine in about 70 % of cases which primary parasitoid species was used as host by the two hyperparasitoid species examined within this study. Here these trophic links between primary and secondary parasitoids (Table 4 in Traugott et al. 2008) are displayed as graphical network. As only one species of cereal aphids was investigated, only the parasitoid network is displayed in the food web. Abundances for primary and secondary parasitoids are available (Table 3 and Table 4 in Traugott et al. 2008) and a one to one relationship exists between primary and secondary parasitoids, i.e. each individual of a secondary parasitoid can be linked to one primary parasitoid and aphid individual, respectively. Thus, the interactions can be quantified and the two trophic levels in the food web share the same scaling. The trophic interactions/parasitisations are displayed as bars and the direction of the trophic links is top-down (Fig. 2).

## Author contribution statement

DS and MT developed the idea and concept for the software, DS developed and implemented the software, DS and MT wrote the manuscript.

## Electronic supplementary material

Supplementary material 1 (PDF 1707 kb)
