# Supplementary material for: Food Web Designer: a flexible tool to visualize interaction networks
Source: J Pest Sci (2004). 2015 Aug 9;89:1–5. doi: 10.1007/s10340-015-0686-7 (PMC4757606; doi:10.1007/s10340-015-0686-7)

Food Web Designer 3.0

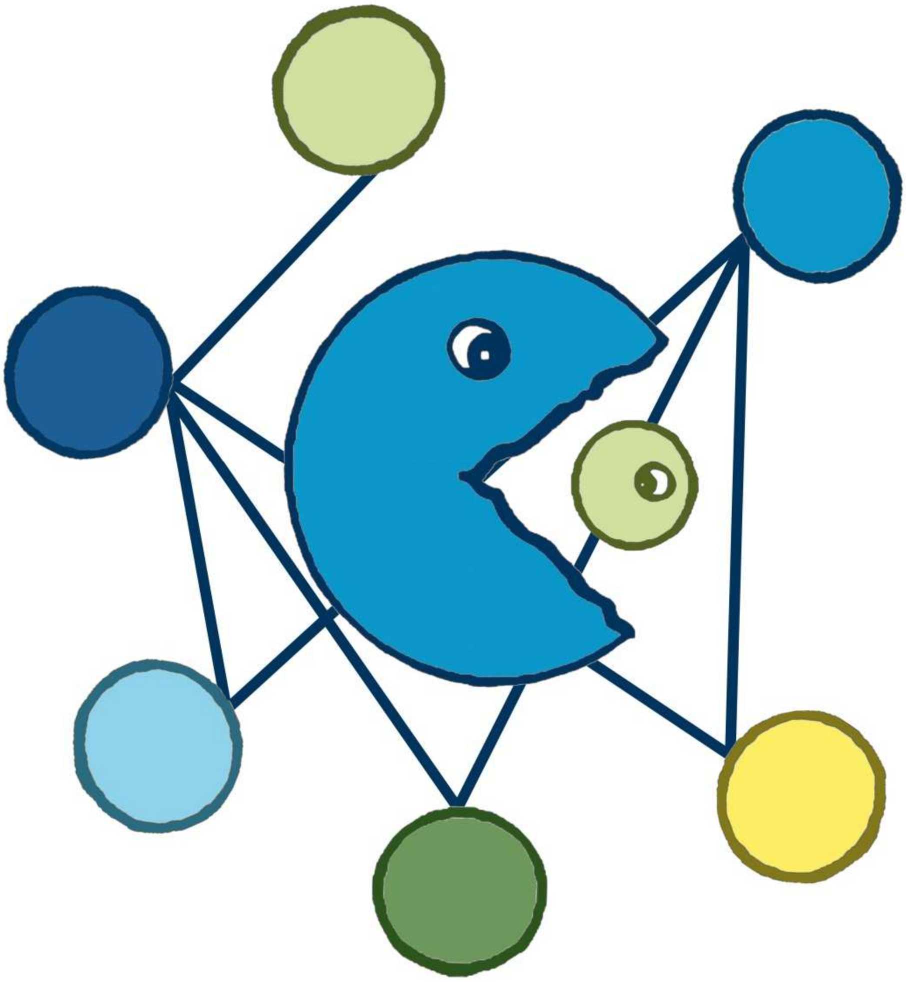

Applied and Trophic Ecology
University of Innsbruck

Food Web Designer v.3.0

Content

General Information ... 2

Installation ... 2

Using Food Web Designer ... 5

Food-web Window – displaying the food-web ... 5

General adjustments ... 6

File ... 6

Window ... 7

Help and Information (?) ... 8

Species Window – handling species data ... 8

Direct data input ... 8

Data import ... 9

Editing species data ... 11

Auto color function ... 11

Deleting species data ... 12

Importing example files ... 12

Interactions Window – handling interaction data ... 13

Direct data input ... 14

Data import ... 16

Editing interaction data ... 16

Deleting interaction data ... 16

Importing example files ... 17

Options Window – adjusting the food-web ... 18

General settings ... 18

Importing example files ... 19

Direction of Links ... 20

Scale ... 20

Legend ... 22

Legend Window – displaying the full legend ... 24

Bugs ... 24

General Information

This help file guides you step-by-step through the process of generating your own food-webs.

Words in CAPITAL letters indicate either the selection of menu entries or clicking on buttons.

Paragraphs in *italic* give detailed instructions on how to to import the example data provided with the Food Web Designer. This example data allows you to generate the very same food-webs presented in this help file.

Installation

Administrator rights may be needed to correctly install Food Web Designer 3.0

Unpack the zip file on your computer.

Double-click on the file 'setup.exe' to start the Food Web Designer Setup Wizard.
If the Microsoft .NET Framework 3.5 or higher is not available on the computer, you will be asked to install it (from the Microsoft web-page) prior to Food Web Designer. To do so, an internet connection might be needed.

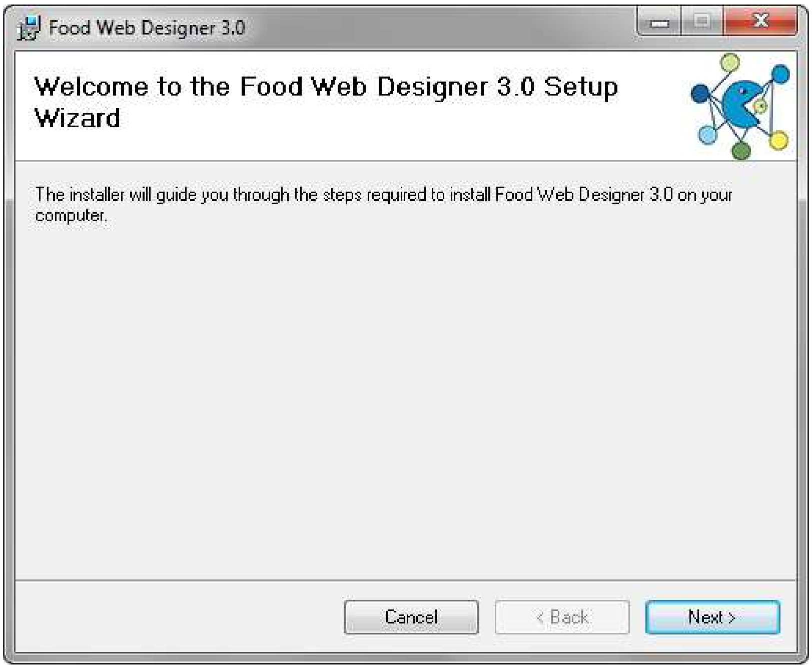

click NEXT

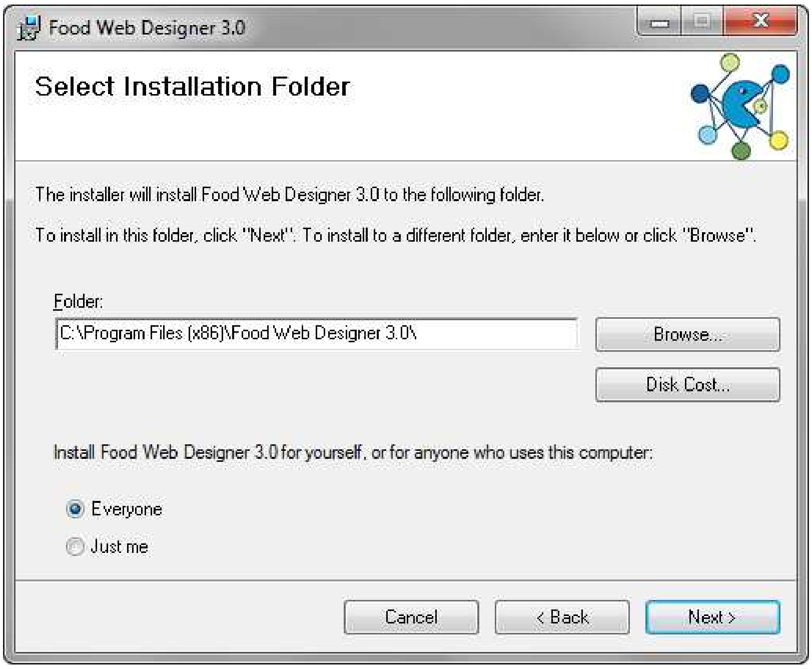

An installation path can be selected on this screen and the decision made who will be able to use the program after installation

click NEXT

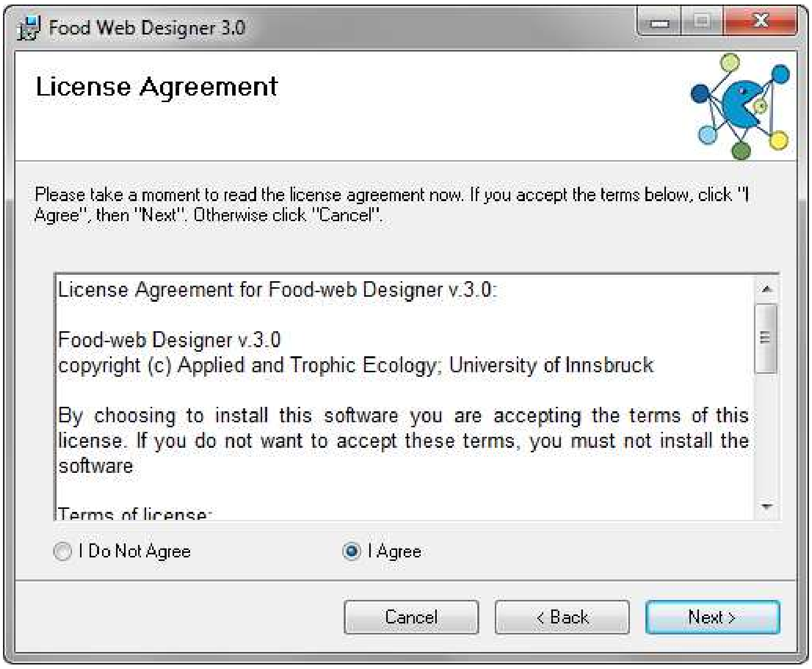

You have to agree to the terms of license if you want to install and use Food Web Designer.

Read the license agreement, select 'I Agree' and click NEXT

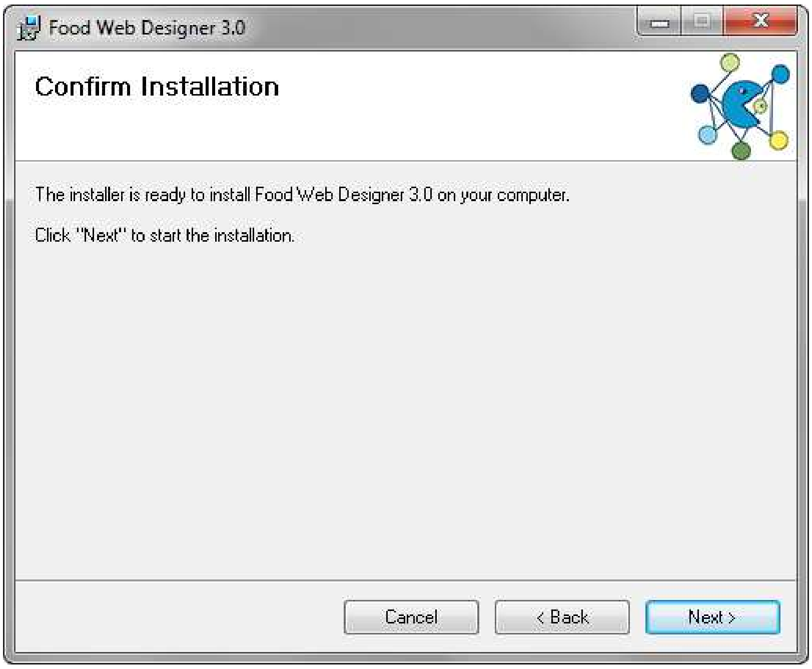

click NEXT to start the installation

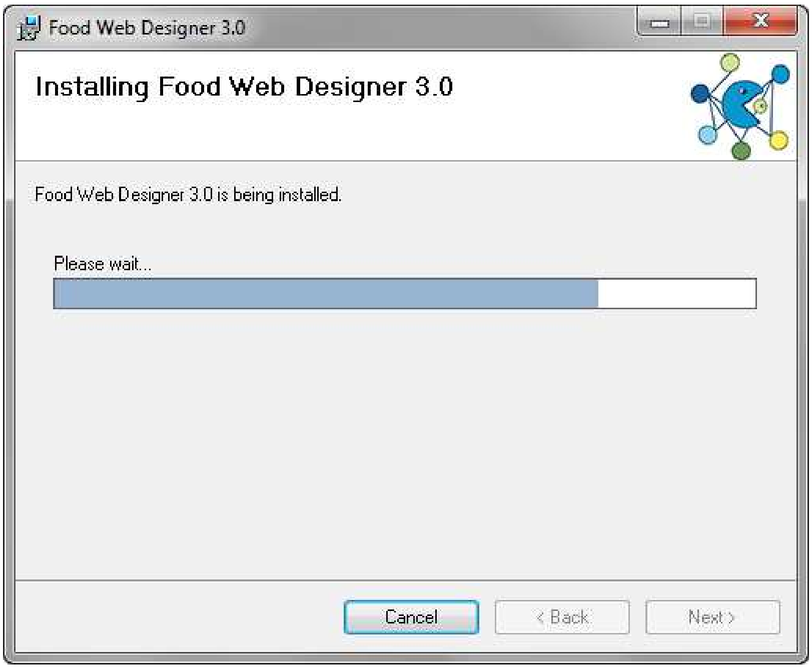

a progress bar is displayed during installation

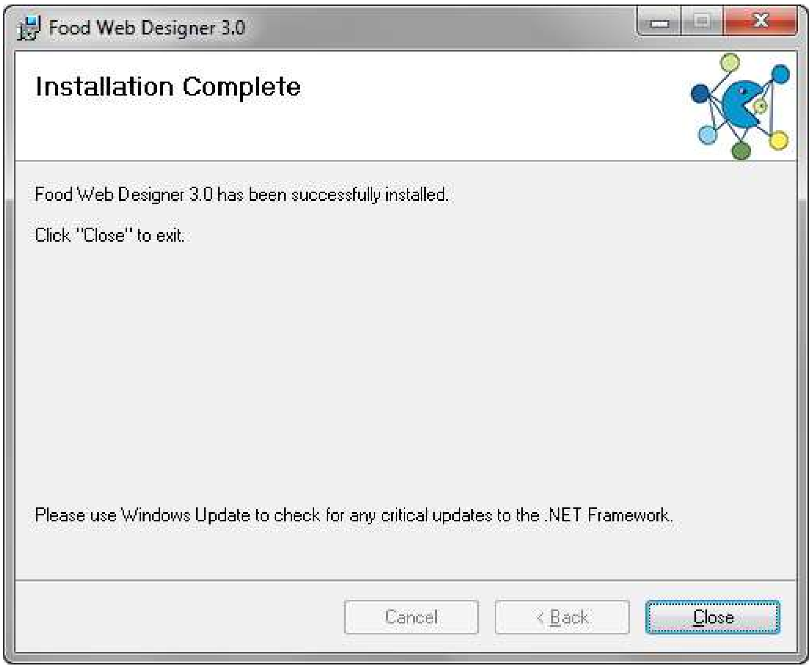

this screen informs you the installation was successful

Using Food Web Designer

Food-web Window – displaying the food-web

After starting the program, the Food-web Window opens.

Once the active food-web project is saved or if an existing food-web project is opened, the file path to the saving location will be displayed in the field '**current project**'. You can also type a file path into this field or change it to the location of where you want to save the current project. However, if you select the **save project** menu entry (see below) any existing file at that location will be overwritten without warning.

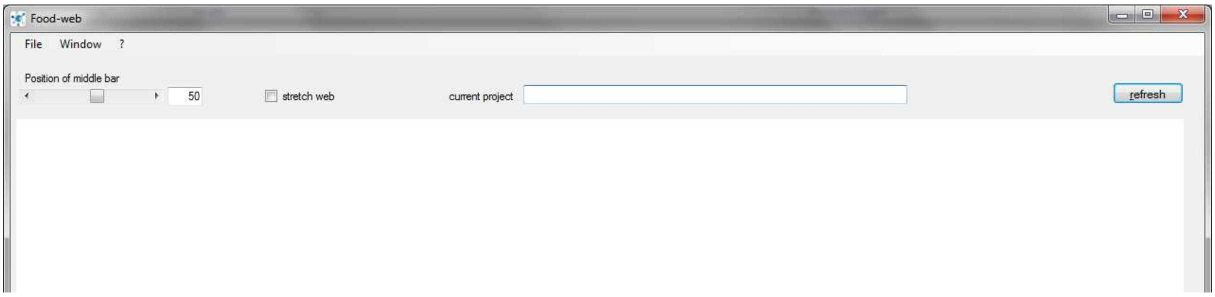

Food-web Window at program start

General adjustments

Adjustments to the "spacing" of the food-web can be made directly in the Food-web Window.

By moving the **scroll bar** the relative position of the middle level, in relation to the top and bottom levels respectively, can be changed.

The **'stretch web'** option increases the vertical distance between the trophic levels.

Options for handling food-web projects (File) and access to windows for data input and handling (Window) can be found in the menu bar. The question mark provides help and information.

File

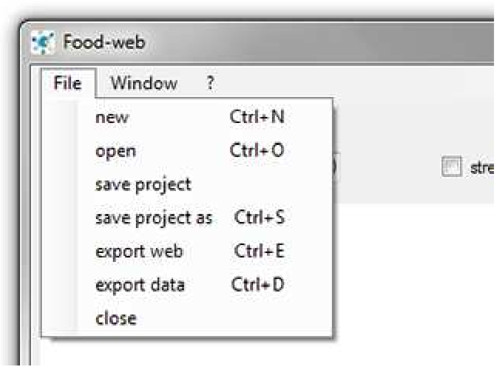

Menu options available when clicking FILE in the Food-web Window

new (Ctrl + N)
: opens a new (empty) food-web project. Only one project can be opened at the time, therefore any unsaved data in the current project will be lost if a new project is opened.

open (Ctrl +O)
: loads an existing food-web project which can be fully edited. Only one project can be opened at the time, therefore any unsaved data in the current project will be lost if another project is opened.

save project
: saves changes in the current food-web project to the file location specified in the 'current project' field — without overwrite warning. If the field is empty, a dialog window opens where you can enter a file name and/or browse to the desired file location.

save project as (Ctrl+S)
: saves the current food-web project under a new name or in a different file location

export web (Ctrl+E)
: saves the current food-web as a picture in either Windows Bitmap format (.bmp) or as Portable Network Graphics (.png)

export data (Ctrl+D)

generates three comma-separated values (.csv) text files (properties.csv, nodes.csv, trophic.links.csv) containing species and interactions data. These files are suitable for direct import into the R package ‘cheddar’ (www.cran.r-project.org/web/packages/cheddar) which can be used for network analysis.

close

exits Food Web Designer. Any unsaved data in the current project will be lost.

Window

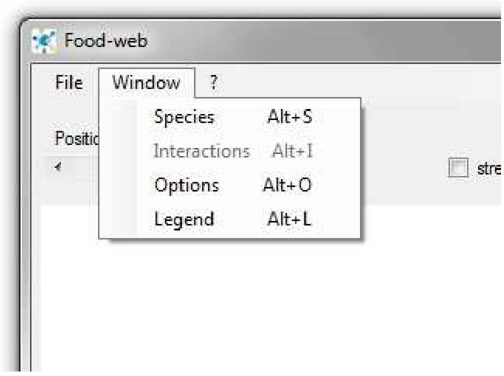

Menu options available when clicking WINDOW in the Food-web Window

species (Alt + S)

opens the Species window for the input and handling of the species data

interactions (Alt + I)

opens the Interactions window for the input and handling of species interaction data. This menu entry will become active (and selectable) when species are present in two adjacent levels of the food-web project in the "species window"

options (Alt + O)

opens the Options window to adjust the appearance of the food-web

legend (Alt + L)

The **legend** option opens a window containing only the legend. This is especially useful for larger webs with so many taxa that the legend cannot be displayed in full length beside the web. The legend can be exported from the legend window.

Help and Information (?)

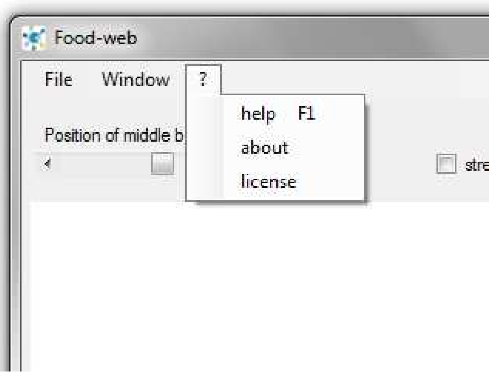

Menu options available when clicking ? in the Food-web Window

help (F1)

opens this help file

about

provides general information on Food-web designer

license

opens the license agreement that was accepted during program installation

Species Window – handling species data

open the Species Window by selecting WINDOW/SPECIES

Species data can be entered by either direct input or an import function.

Direct data input

Species data can be entered in the corresponding fields of the input section of the species window. Clicking on the COLOR button will open a color dialog. A pattern can be selected from the drop-down list.

By clicking the button ADD TO … LEVEL, the entered species is added with the selected properties to the top, middle or bottom level of the food-web.

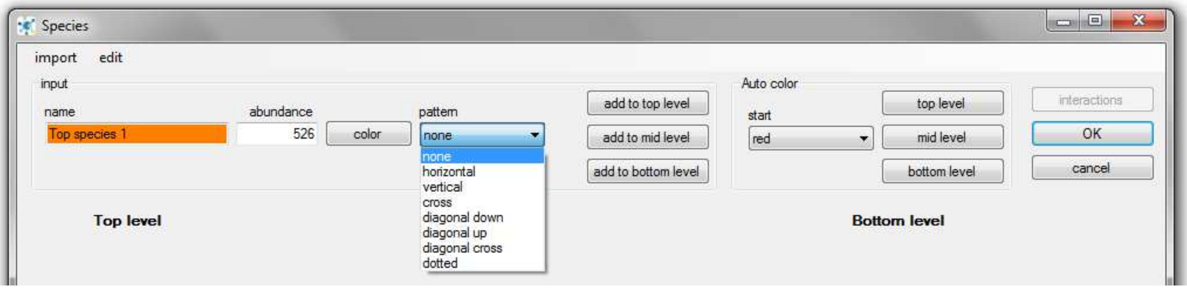

Species Window during direct input of species data

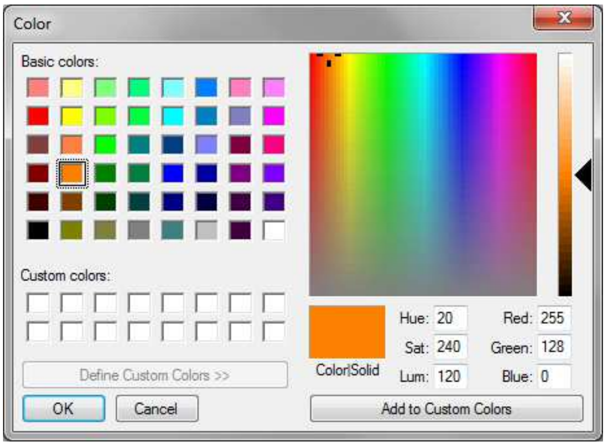

Dialog window for selecting colors

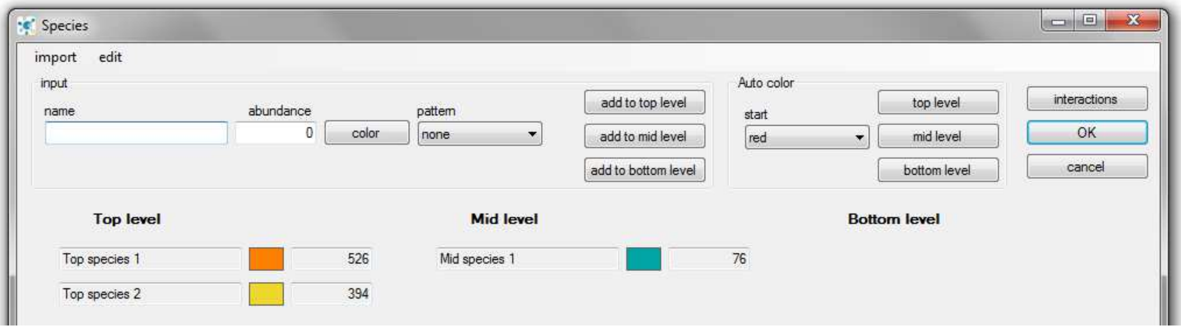

Species Window with 3 species entered to the food-web project

Data import

Larger sets of species, where direct entry might not be convenient, can easily be imported via the **IMPORT** menu. When selecting IMPORT/TOP LEVEL a dialog window will open where you can select the file containing your species data. The same applies to MID LEVEL and BOTTOM LEVEL.

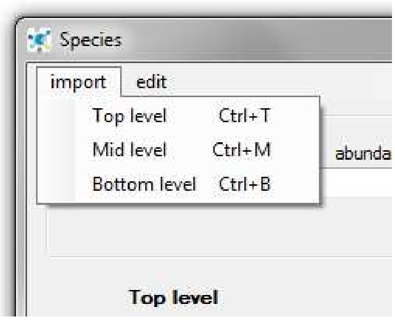

Menu options available when clicking IMPORT in the Species Window

The import function reads semicolon (;) delimited text files (*.csv) with the following specifications:

- NO headers in the table
- The first column is mandatory and contains the species name. All other columns are optional. Imported data can be edited at any time in the species window.
- The second column can contain the abundance. If it is empty, missing or cannot be interpreted correctly, an abundance of 0 (zero) is assigned.
- The third column can contain a color to be assigned to the species. Colors which are recognized by name are listed below. Alternatively (A)RGB values can be provided comma (,) separated or as signed integer. If no color is specified or recognized, color "Gray" is assigned.
- The fourth column can contain a pattern. All entries from the drop-down list are recognized. If no pattern is specified or recognized, no pattern is assigned to the species.

Important notes:
If csv-files are generated using Microsoft Excel, depending on the regional and language settings, a comma (,) might be used as separator instead of a semicolon (;). The separator of an existing csv-file can be changed using the replace function of a text editor.
Importing data to a trophic level which already contains some species will not extend the species list but will replace it! Imported species data can be extended by direct entry.

Examples for species import data can be found in the program folder in "example data": species_bottom-names.csv, species_mid-RGB.csv, species_top-RGB-integer.csv

AliceBlue	DarkSlateGray	LightSalmon	PaleVioletRed
AntiqueWhite	DarkTurquoise	LightSeaGreen	PapayaWhip
Aqua	DarkViolet	LightSkyBlue	PeachPuff
Aquamarine	DeepPink	LightSlateGray	Peru
Azure	DeepSkyBlue	LightSteelBlue	Pink
Beige	DimGray	LightYellow	Plum
Bisque	DodgerBlue	Lime	PowderBlue
Black	Firebrick	LimeGreen	Purple
BlanchedAlmond	FloralWhite	Linen	Red
Blue	ForestGreen	Magenta	RosyBrown
BlueViolet	Fuchsia	Maroon	RoyalBlue
Brown	Gainsboro	MediumAquamarine	SaddleBrown
BurlyWood	GhostWhite	MediumBlue	Salmon
CadetBlue	Gold	MediumOrchid	SandyBrown
Chartreuse	Goldenrod	MediumPurple	SeaGreen
Chocolate	Gray	MediumSeaGreen	SeaShell
Coral	Green	MediumSlateBlue	Sienna
CornflowerBlue	GreenYellow	MediumSpringGreen	Silver
Cornsilk	Honeydew	MediumTurquoise	SkyBlue
Crimson	HotPink	MediumVioletRed	SlateBlue
Cyan	IndianRed	MidnightBlue	SlateGray
DarkBlue	Indigo	MintCream	Snow
DarkCyan	Ivory	MistyRose	SpringGreen
DarkGoldenrod	Khaki	Moccasin	SteelBlue
DarkGray	Lavender	NavajoWhite	Tan
DarkGreen	LavenderBlush	Navy	Teal
DarkKhaki	LawnGreen	OldLace	Thistle
DarkMagenta	LemonChiffon	Olive	Tomato
DarkOliveGreen	LightBlue	OliveDrab	Turquoise
DarkOrange	LightCoral	Orange	Violet
DarkOrchid	LightCyan	OrangeRed	Wheat
DarkRed	LightGoldenrodYellow	Orchid	White
DarkSalmon	LightGray	PaleGoldenrod	WhiteSmoke
DarkSeaGreen	LightGreen	PaleGreen	Yellow
DarkSlateBlue	LightPink	PaleTurquoise	YellowGreen

List of all recognized names for colors to be provided in an import file

Editing species data

The Editing Window for species data can be opened via the **EDIT** menu option or by double-clicking the species name.

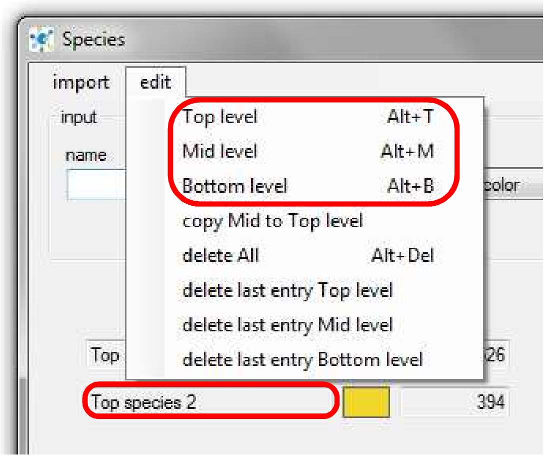

Menu options available when clicking EDIT in the Species Window
Two options are available to open the Editing Window for species data

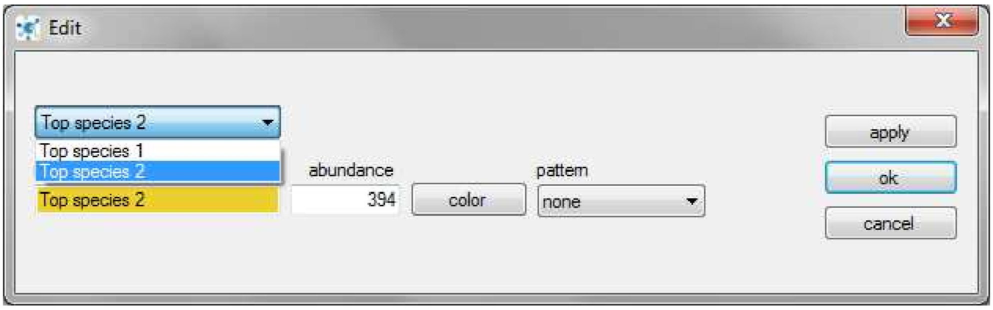

Screenshot of the Editing Window

If several species from the same level need editing, changes can be made via the **APPLY** button, while the editing window stays open. Clicking the **OK** button will change the data and close the editing window. **CANCEL** will close the window without changing the data. However, changes that have been made (by clicking apply) will not be reversed.

Auto color function

The option to automatically assign colors to all species in a level is available by clicking the respective button. 70 colors are preset and the color to start with can be selected from the drop-down list.

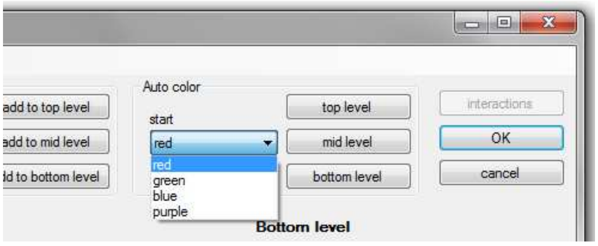

Species Window with open drop-down list for automatic color assignment

Deleting species data

Two options are available to delete species data from the food-web project.

EDIT/DELETE ALL (ALT + Del) will delete all species data from all three levels of the project

EDIT/DELETE LAST ENTRY … will only remove the bottom-most species in the list from the respective trophic level

Importing example files

Select IMPORT/TOP LEVEL from the menu and choose the file "species_top-RGB-integer.csv" from the "example_data" folder in the program folder. The location of this folder depends on the path selected when the program was installed. Import the species information for the mid-level (species_mid-RGB.csv) and bottom level (species_bottom-names.csv) of the web the same way. After clicking on OK the following will be displayed

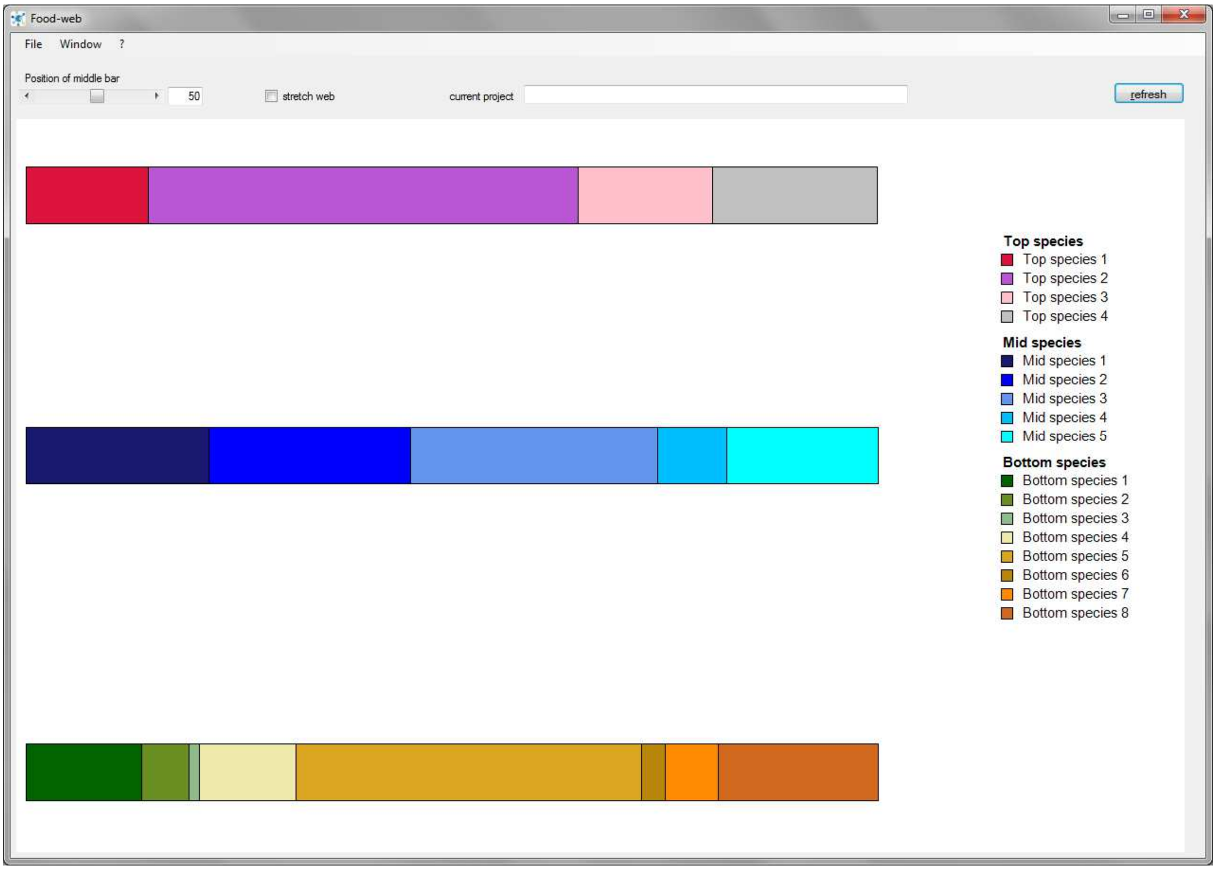

Screenshot of the food-web example when only species data is available.

Interactions Window – handling interaction data

The Interactions Window can only be accessed once species are present in two adjacent levels of the food-web. The Interactions Window can be opened via clicking the **INTERACTIONS** button (Species Window) or the menu option **WINDOW/INTERACTIONS** (Food-web Window).

When opening the Interactions Window for the first time, all possible interactions are listed for the two panels and the strength of each interaction is 0.

The direction of links ('bottom-up' or 'top-down') can be set for both panels separately. It is possible to switch between the two options without losing the respective data from either option.
If 'top-down' is selected, the interactions in the top panel will be Top species → Mid species (where Top species is the consumer and Mid species the resource); otherwise the direction will be Mid species → Top species. The same applies for the bottom panel.

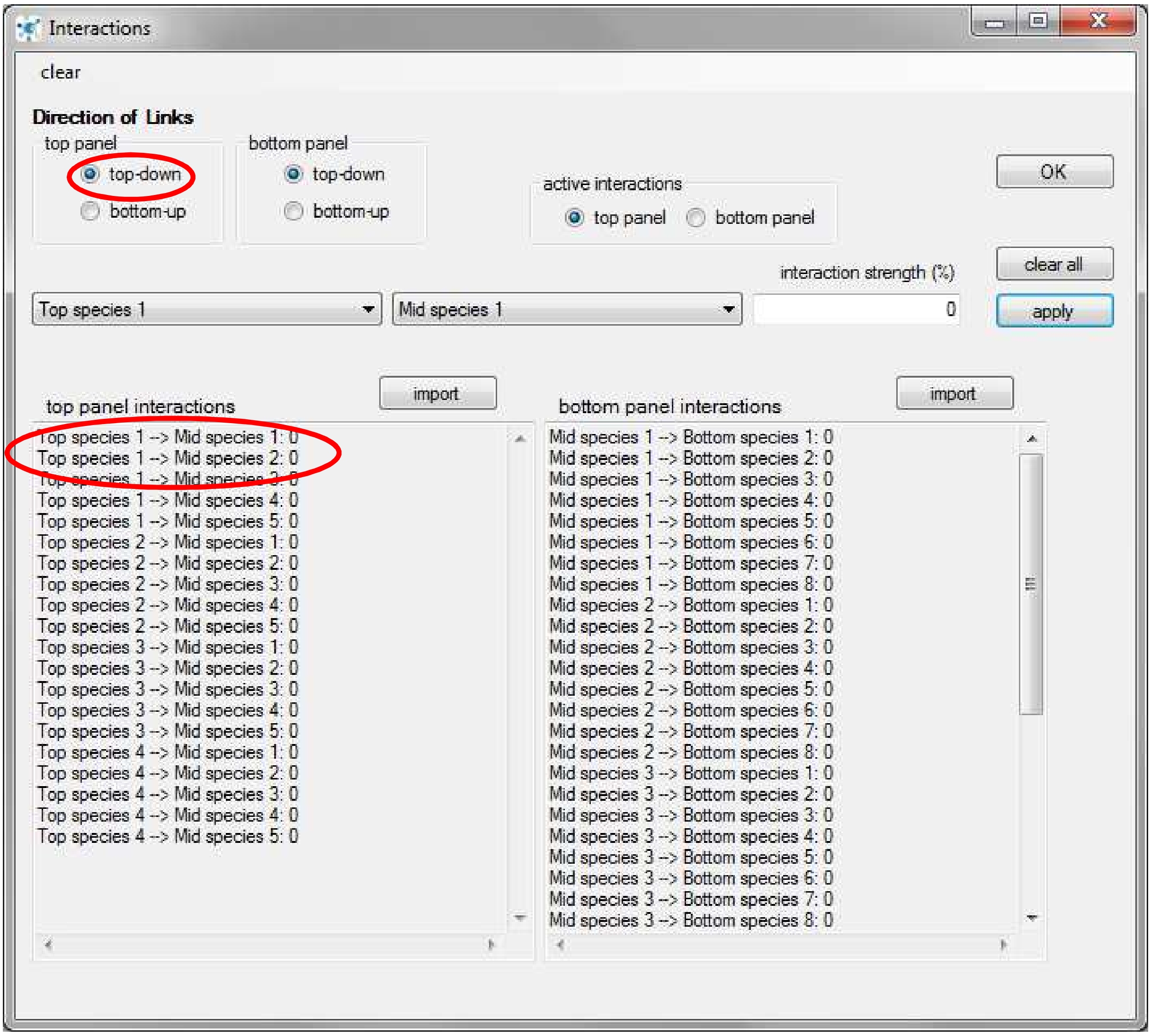

Interactions Window and direction of links with the option 'top-down' selected for the top panel

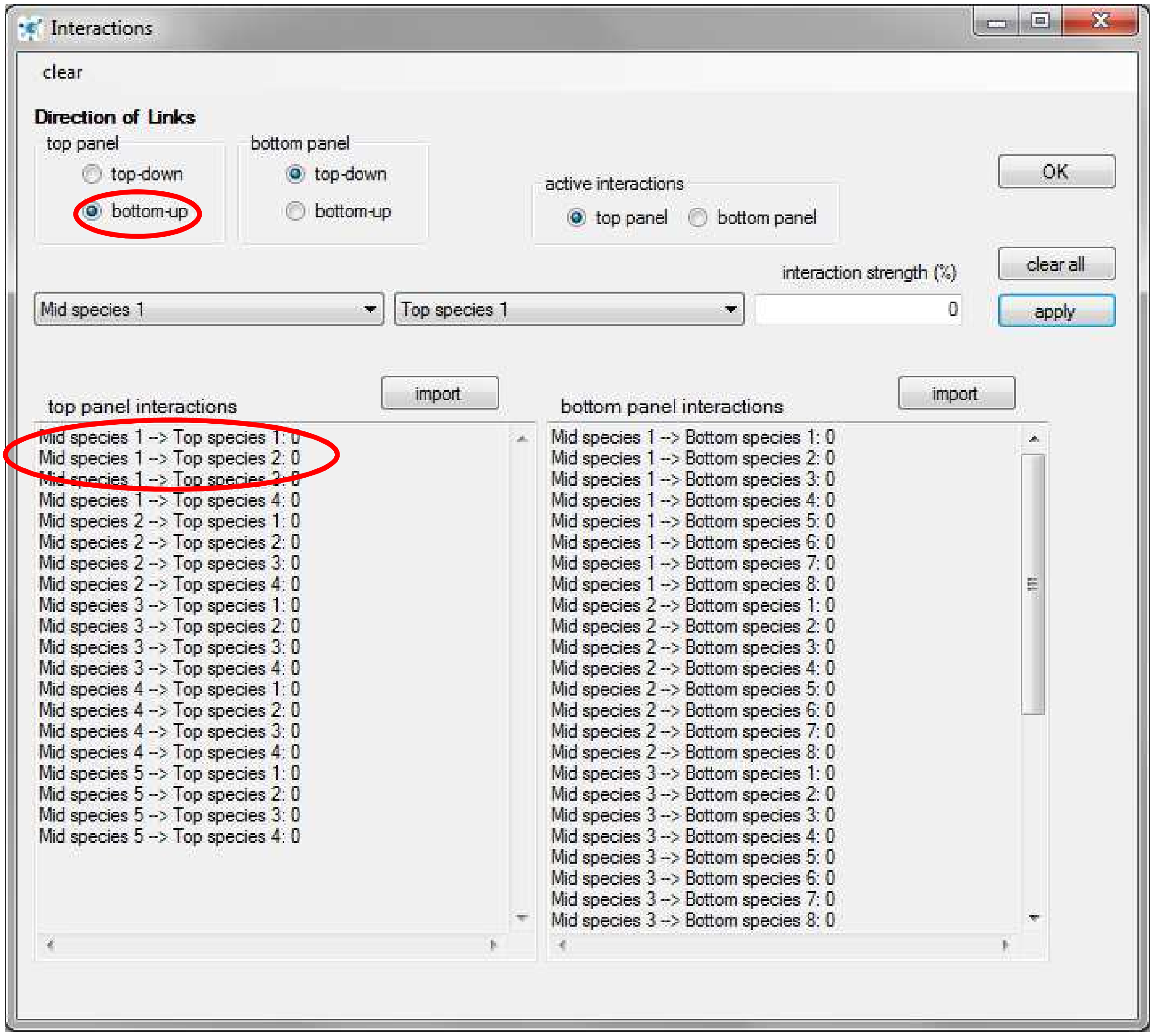

Interactions Window and direction of links with the option 'bottom-up' selected for the top panel

Direct data input

This entry method will be most suited for very small webs or for the correcting / editing of single entries.

- select the direction of links
- select the panel you want to edit in the 'active interactions' box
- select the interacting species from the drop-down lists
- enter the interaction strength in %. The width (basis) of the interaction link will be displayed as the percentage of the corresponding abundance of this species. Therefore, ensure the sum of all interactions of one species does not exceed 100% within one panel. Otherwise the web will not be displayed correctly! A warning message will be shown to you in this case.
- accept the entry by clicking the **APPLY** button

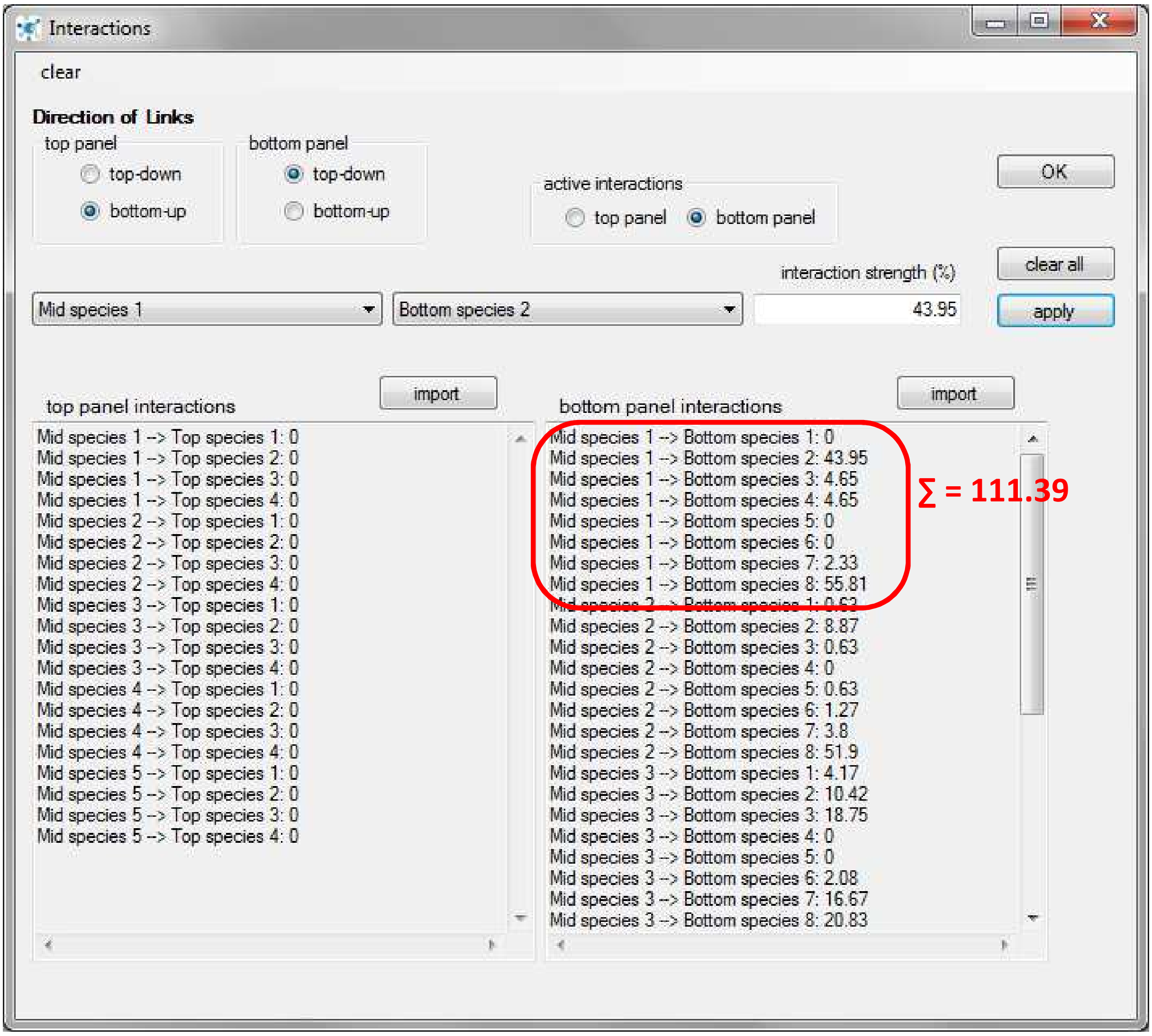

Example in which the total interaction strength of a species exceeds 100%

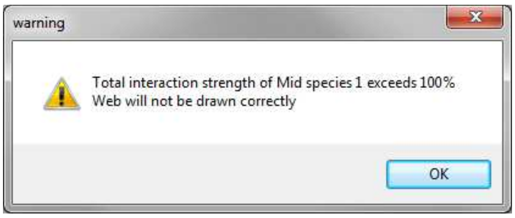

Warning which is displayed for this example

Data import

Alternatively to the direct entry method, the interaction data can be imported from semicolon (;) delimited text files (*.csv). The format is an M x N matrix of the interaction strength without headers with M consumers (rows) and N resources (columns). E.g. a matrix for 3 predators feeding on 4 prey types will have 3 rows and 4 columns. Examples for such files can be found in the program folder "example data"; e.g. "interactions_top_topdown".

Click on the **IMPORT** button above the panel for which you want to import data (check that the correct direction for the links is selected) and select the file containing the interaction data in the import dialog.

If a file with inappropriate data is selected (e.g. the number of rows and columns does not correspond with the number of species in the respective levels), or the file is in use by another program, an error message will be displayed.

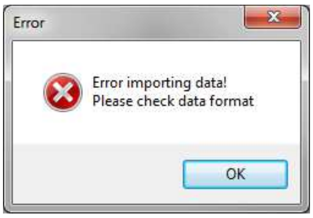

Error message if the interaction data cannot be imported correctly

Editing interaction data

To edit single or few interactions, direct data input is the most suitable method(see above).

To edit many interactions in complex networks, it is easiest to generate / correct the import file and re-import the full interaction table via the import function (see above).

Deleting interaction data

Via the **CLEAR** menu on the interactions window all interactions of either the **top panel**, the **bottom panel** or **all panels** can be set to zero. While clearing top or bottom panel will only reset the respective interactions table (depending on the direction of links), both versions will be set to zero when selecting all panels or by clicking the **CLEAR ALL** button.

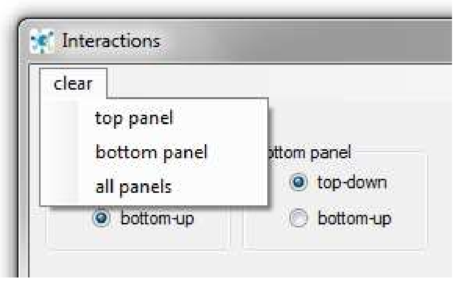

Menu options available when clicking CLEAR in the Interactions Window

Importing example files

Be sure the example species were imported to your food-web project (see above). Then select 'TOP-DOWN' for the top panel and click the IMPORT button above the field showing the top panel interactions (left side). Browse to the "example_data" folder in the program folder, select "interactions_top_topdown.csv" and click OPEN. Change the direction of links to 'BOTTOM-UP' and import the file "interactions_top_bottomup.csv" to the top panel. Repeat the whole procedure for the bottom panel (right side) and import "interactions_bottom_topdown.csv" and "interactions_bottom_bottomup.csv" respectively.

As the example contains different numbers of species in the top level (4 species) and mid-level (5 species) of the web, the interaction matrix has a 4 x 5 format for the top-down links and a 5 x 4 format for the bottom-up links. You can check this by opening the respective csv-files with a text editor or a table calculation program. The same applies for the bottom level.

After clicking OK the complete web will be displayed. If the web was not updated automatically, click the REFRESH button on the Food-web Window. The direction of the displayed links depends on the currently selected options in the Interactions window.

Alternatively, this full food-web project containing species and interaction data can be opened via FILE/OPEN and selecting 'example-web_triangles.xml' from the "example_data" folder.

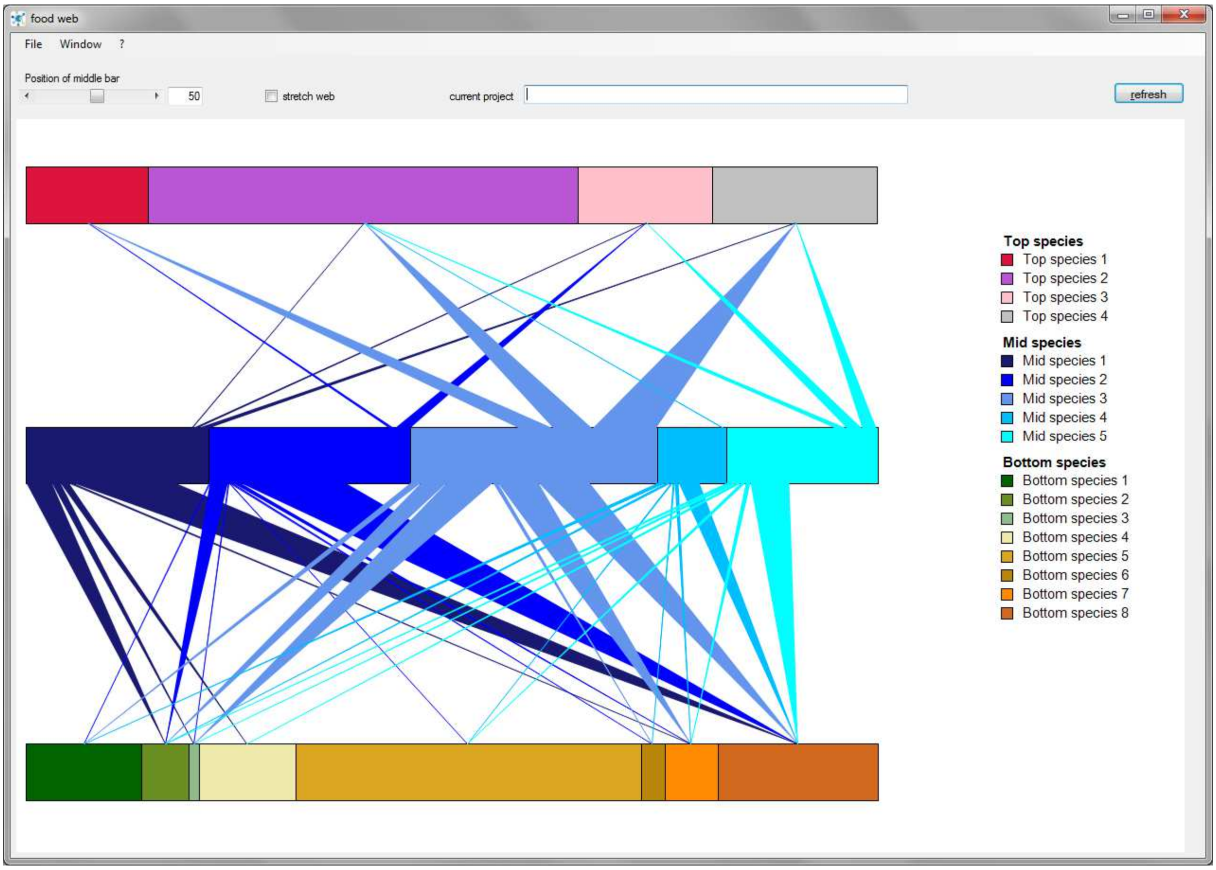

Screenshot of the food-web example when species and interaction data is available. For this web the option 'bottom-up' was selected for the direction of links in the top panel and 'top-down' for the bottom panel.

Options Window – adjusting the food-web

Most adjustments can be made in the Options Window which is opened via the menu option **WINDOW/OPTIONS** or the shortcut Alt + O.

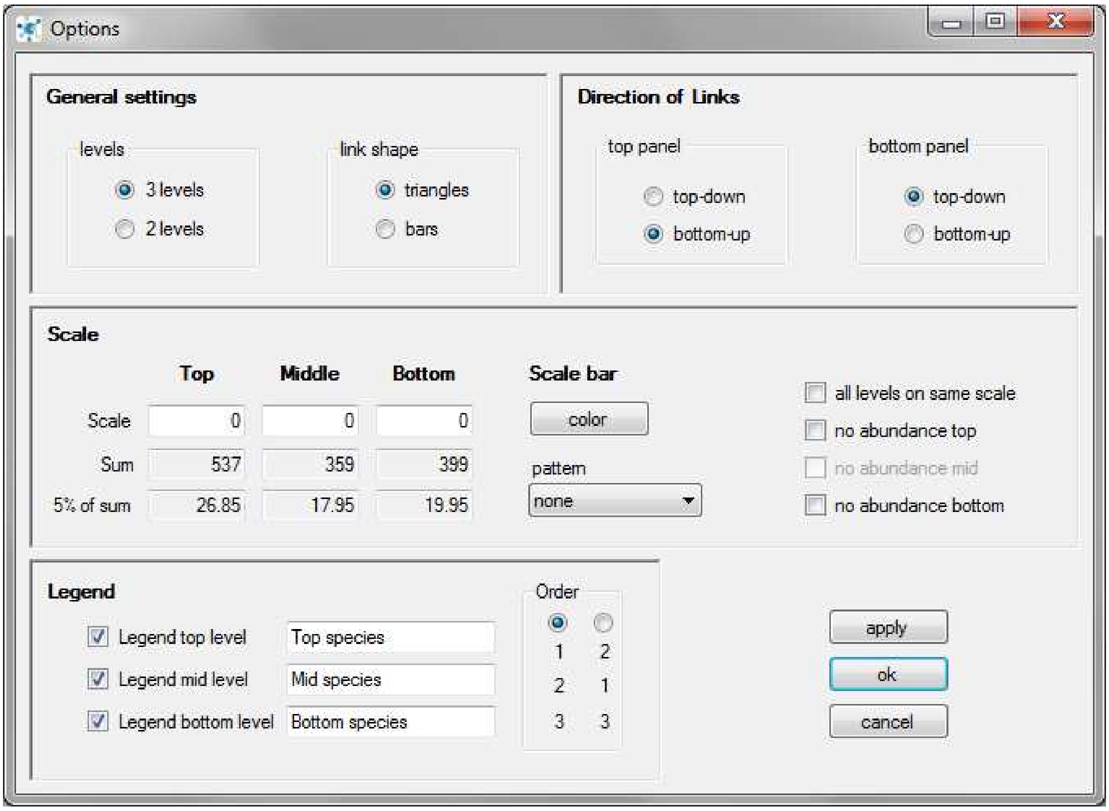

Options Window

Changes that are made via the **APPLY** button allow you to check the new appearance of the food-web, while the Options Window stays open. Clicking the **OK** button will apply the changes and close the Options Window. **CANCEL** will close the Options Window without changes; however, changes that have already been made will not be reversed.

General settings

In the General settings section, you can define whether the food-web consists of two or three interacting levels. If **'2 levels'** is selected, the top panel will not be displayed in the web (but no data will be lost). Also the shape of the links (triangles or bars) can be changed here.

Important information if links are displayed as bars:
In this case, the data from both interaction tables (top-down and bottom-up) is combined. The interaction strength from each table defines the width of the bar at the side of the respective species (corresponding to the consumer in each table).

In this way it is possible to draw bars with parallel sides but also trapezoid shaped bars.

For bars with parallel sides, the option **'all levels on same scale'** (see below) has to be selected in the Options Window to ensure each individual takes the same space in both levels. Furthermore the

interaction strength has to be calculated to cover the correct percentage for both interacting species. e.g. species 1 (middle level) has an abundance of 20 individuals and species 2 (bottom level) 100 individuals. If 10 individuals are interacting, the percentage for the interaction strength has to be 50 in the top-down table (middle level → bottom level) and 10 in the bottom-up table (bottom level → middle level) because 10 individuals is 50% of the species 1 population but 10% of the species 2 population.

Importing example files

Be sure the example species were imported to your food-web project (see above). Then select 'TOP-DOWN' for the top panel and click the ***IMPORT*** *button above the field showing the top panel interactions (left side). Browse to the "example_data" folder in the program folder, select "bars_top_topdown.csv" and click OPEN. Change the direction of links to 'BOTTOM-UP' and import the file "bars_top_bottomup.csv" to the top panel. Repeat the whole procedure for the bottom panel (right side) and import "bars_bottom_topdown.csv" and "bars_bottom_bottomup.csv" respectively.*

After clicking OK the web will be displayed. If the web was not updated automatically, click on the ***REFRESH*** *button on the Food-web Window. Open the Options Window (WINDOW/OPTIONS) and select* ***'bars'*** *as link shape. Apply the changes by clicking APPLY or OK. The color of the displayed links depends on the direction of links currently selected.*

Alternatively, this full food-web project containing species and interaction data can be opened via FILE/OPEN and selecting 'example-web_bars.xml' from the "example_data" folder.

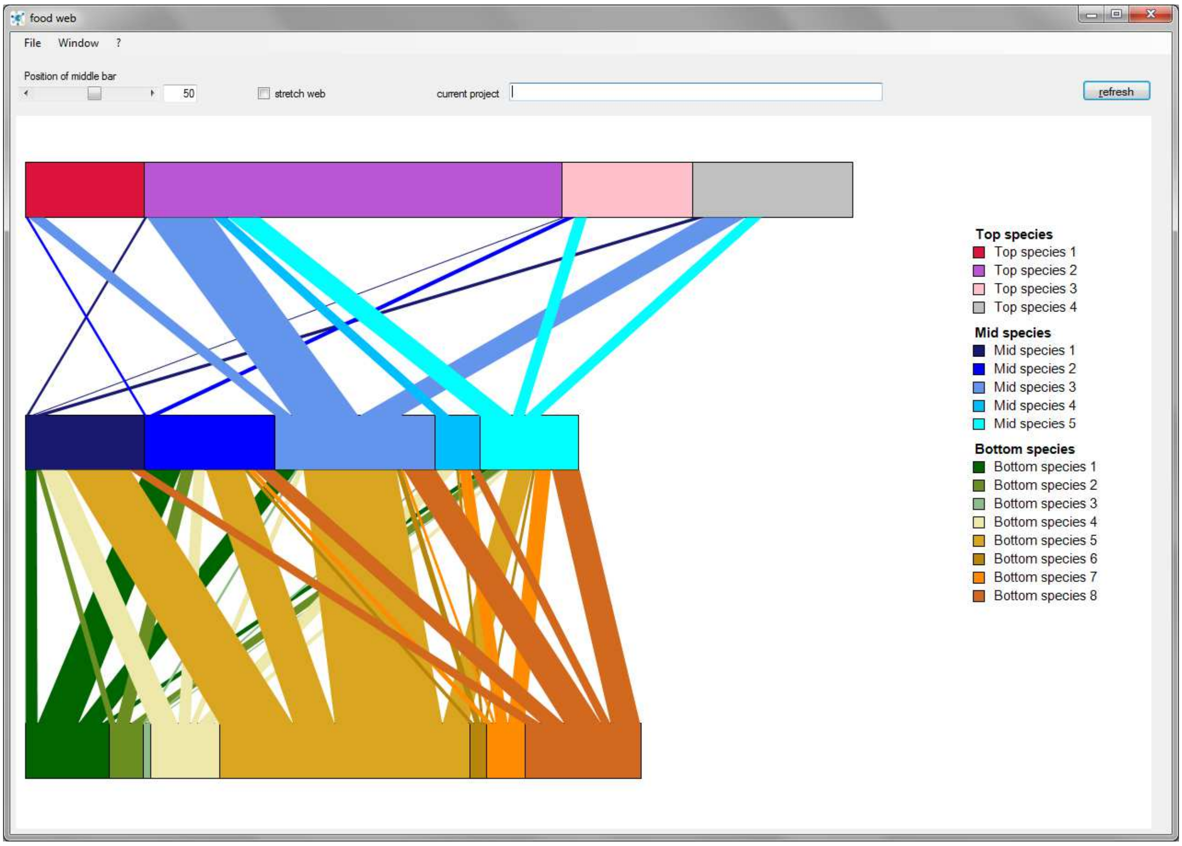

Screenshot of the food-web example with bars as link shape. For this web the option 'bottom-up' was selected for the direction of links in both panels.

Direction of Links

In the Direction of Links section you can switch between top-down and bottom-up interactions. **Note:** the color of the links corresponds to the color of the consumer; this also applies if bars are selected as link shape.

Scale

The option **'all levels on same scale'** defines and adjusts the scaling across the different levels. If this option is selected, the level with the highest total abundance will be displayed in full width and the other levels with a lower abundance will be compressed relative to their total abundance. In this way one individual covers the same width in every level.

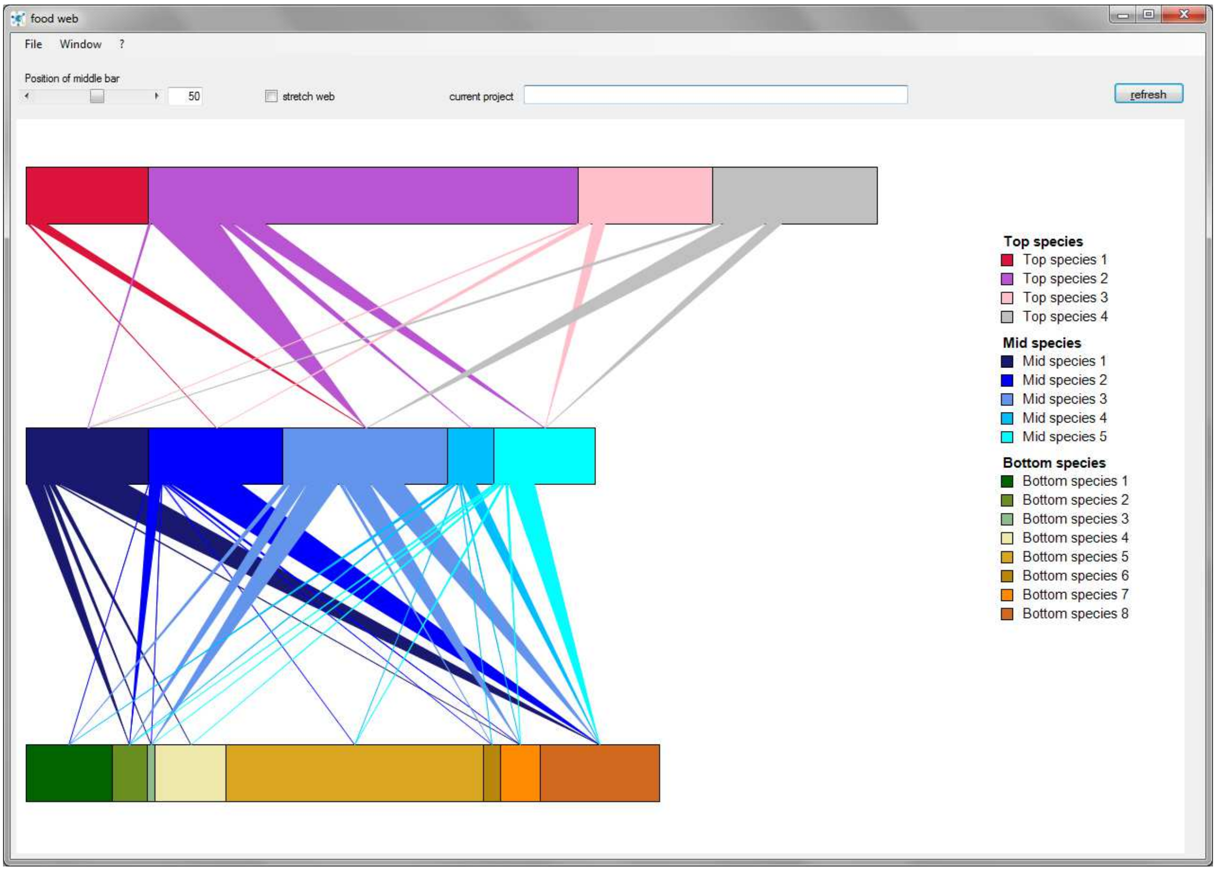

Screenshot of the food-web example if the option 'all levels on same scale' is selected. For this web the option 'top down' was selected for the direction of links in both panels.

For levels which serve only as resources, the option **'no abundance'** can be selected to display the species as circles without abundance dimension instead of the standard bars. This option is not available if links are displayed as bars.

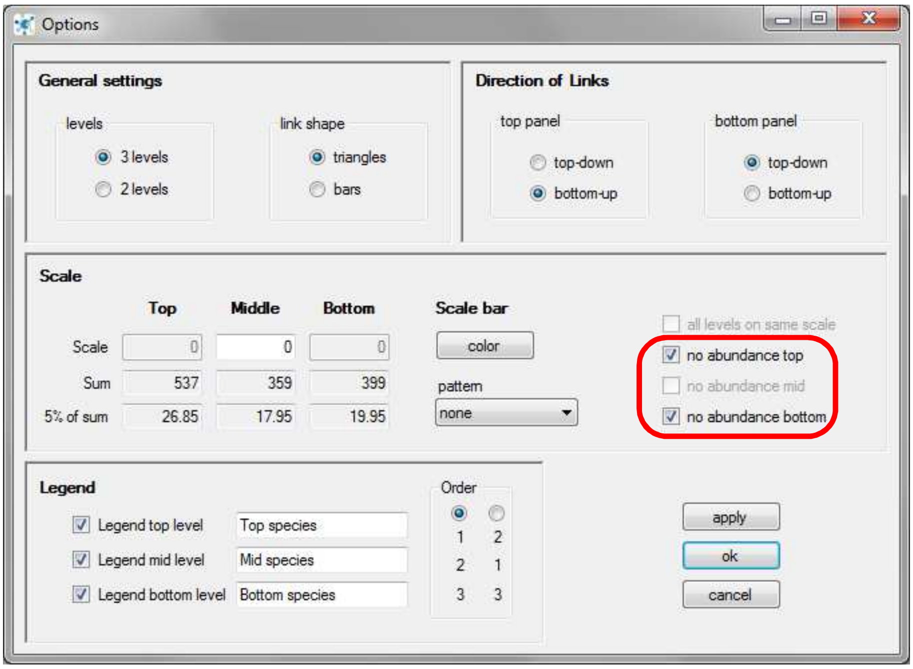

Options Window with 'no abundance' selected for the top and bottom level

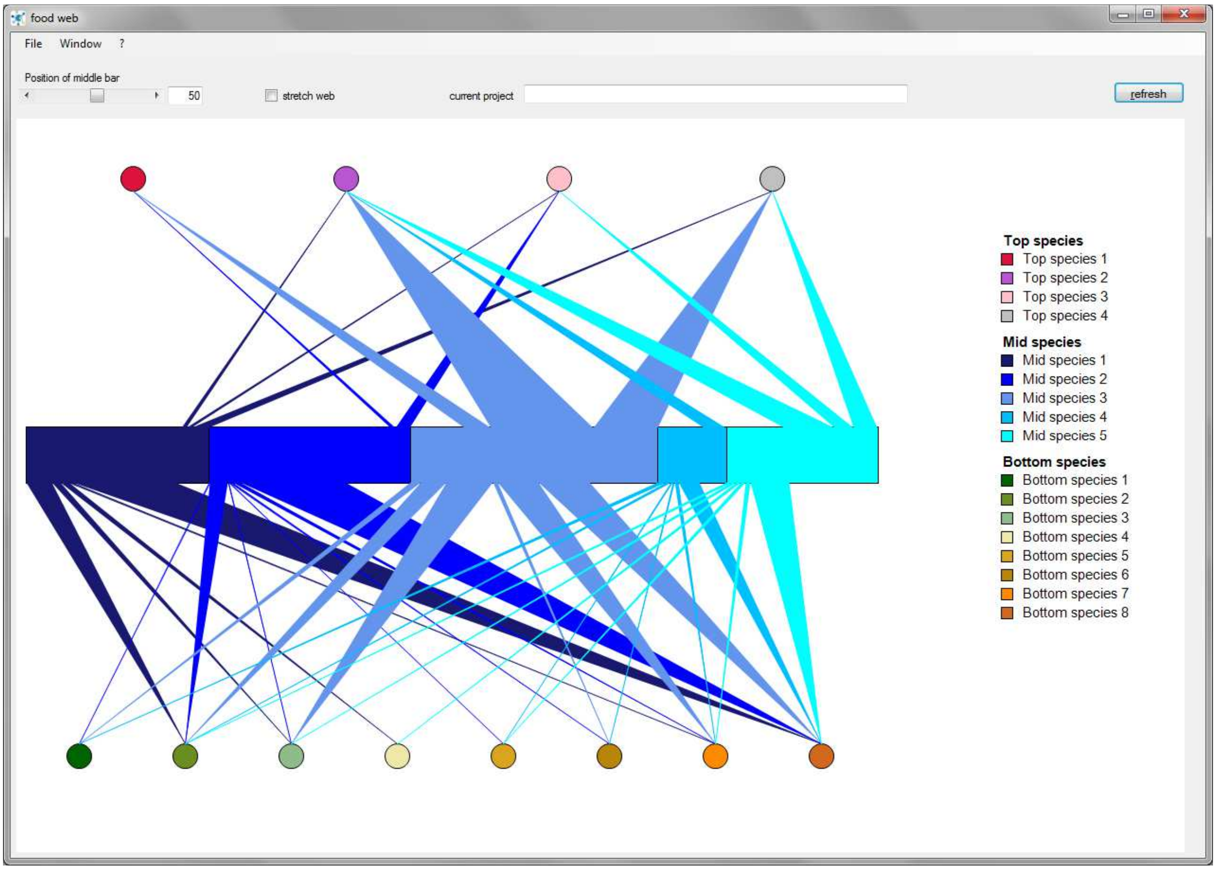

Screenshot of the food-web example if the option 'no abundance' is selected for the top and bottom level

Beside each bar representing a trophic level a **scaling bar** for the abundances can be added. In the Sum field, the total abundance for each trophic level is displayed. The 5% of the total abundance for each trophic level (5% of sum) might serve as an orientation for which number to enter in the Scale field(s).

If all trophic levels are displayed at the same scale, only the scale for the level with the highest abundance will be available.

Color and pattern of the scaling bar can be adjusted by selecting the appropriate information from the color dialog and the drop down list in the Options Window.

To remove the scale bar from a certain trophic level, re-set the value to zero.

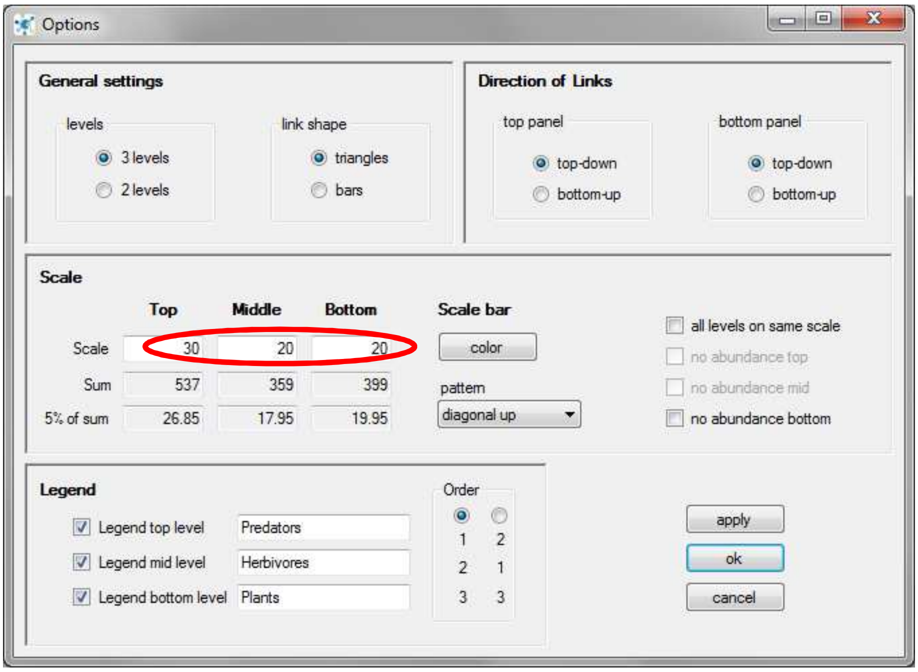

Options Window with set values to be displayed as scale beside each trophic level

Legend

The legend for each trophic level can be included / excluded separately by selecting the appropriate check boxes.

The heading for each level can be changed by typing into the corresponding field.

In the Order box the display order of the legend can be selected for the different levels

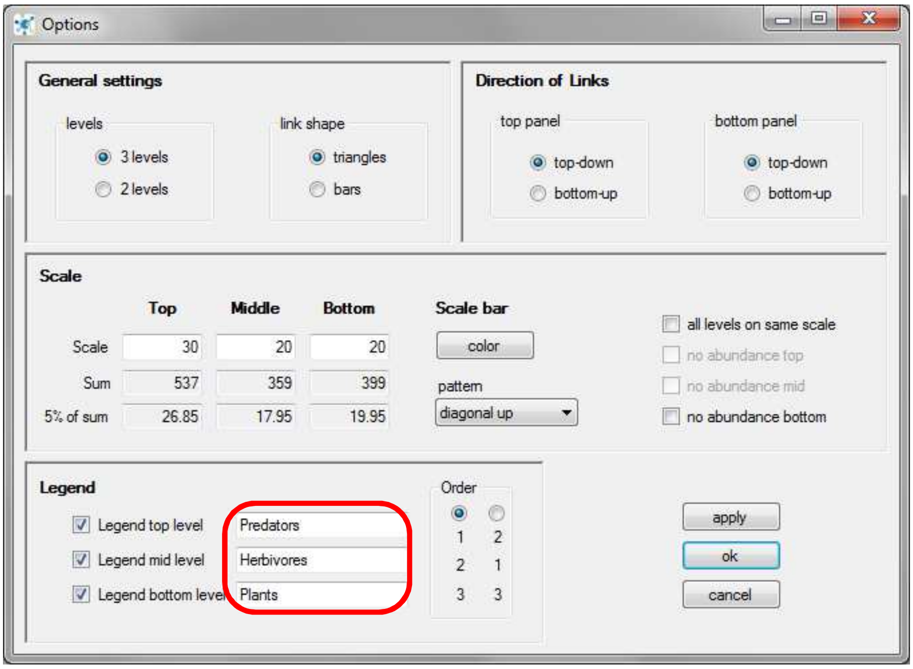

Options Window with set names for trophic levels

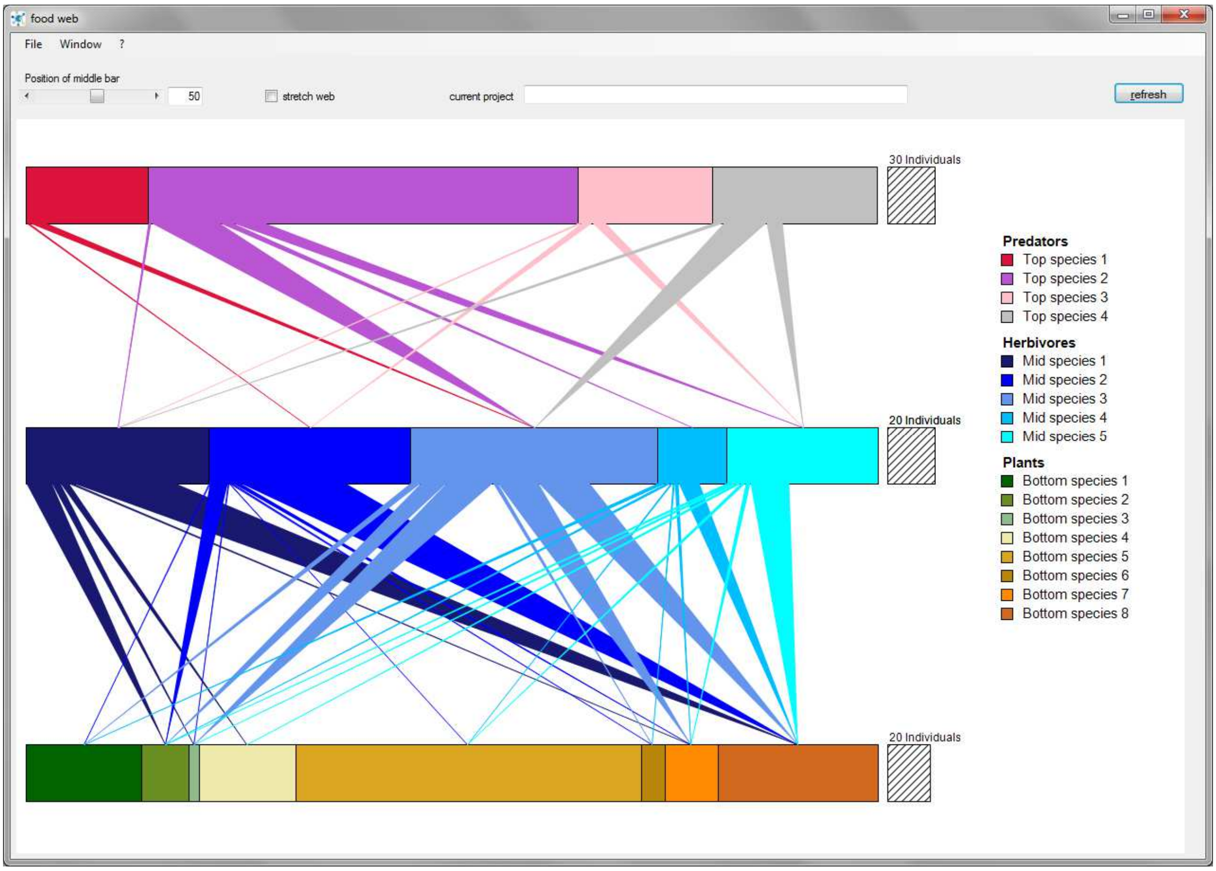

Screenshot of the food-web example with scale bars displayed and changed legend headings for the trophic levels

Legend Window – displaying the full legend

The Legend window contains only the legend and can be opened via the menu option WINDOW/LEGEND in the Food-web Window. Clicking EXPORT in the Legend Window will allow you to save the legend in either Windows Bitmap format (.bmp) or as Portable Netword Graphics (.png).

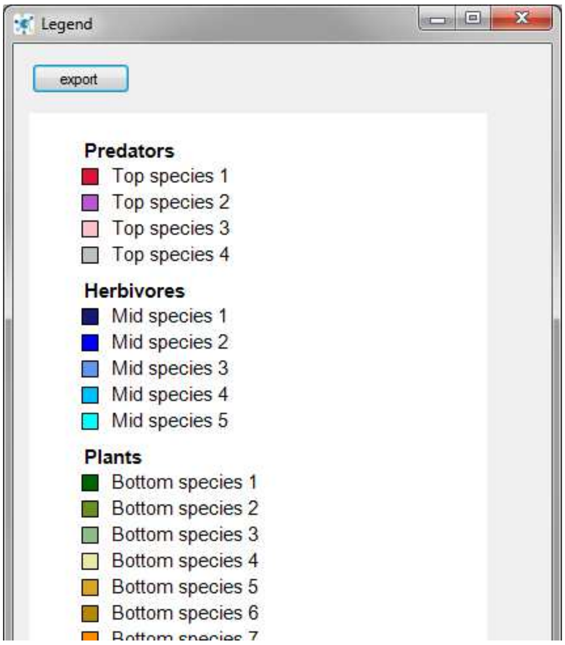

Legend Window

Bugs

As entomologists we are experienced in catching bugs and we did our best to find all the hidden ones in this program. However, we will have missed one or the other…
We would be grateful if you report them (or suggestions on how to improve the program) to us

Daniela.Sint@uibk.ac.at

Michael.Traugott@uibk.ac.at

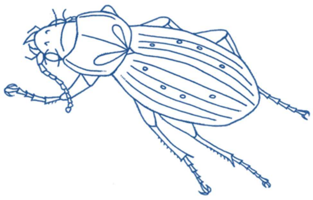

Supplement: Supplementary file 1 — Supplementary material 1 (PDF 1707 kb) [file 10340_2015_686_MOESM1_ESM.pdf]
